# Impacting clinical evaluation of anterior talofibular ligament injuries through analysis of ultrasound images

**DOI:** 10.1186/s12938-016-0129-6

**Published:** 2016-02-02

**Authors:** Vedpal Singh, Irraivan Elamvazuthi, Varun Jeoti, John George, Akshya Swain, Dileep Kumar

**Affiliations:** Centre for Intelligent Signal and Imaging Research (CISIR), Department of Electrical and Electronic Engineering, Universiti Teknologi PETRONAS, Bandar Seri Iskandar, 32610 Perak Darul Ridzuan Malaysia; Research Imaging Centre, University of Malaya, Kuala Lumpur, 50603 Malaysia; Department of Electrical and Computer Engineering, University of Auckland, Private Bag 92019, Auckland, 1142 New Zealand

**Keywords:** Anterior talofibular ligament, Ultrasound imaging, Segmentation, Particle swarm optimization, Adaptive histogram equalization, Curve evolution, Morphological operation, Validation metrics

## Abstract

**Background:**

Anterior talofibular ligament (ATFL) is considered as the weakest ankle ligament that is most prone to injuries. Ultrasound imaging with its portable, non-invasive and non-ionizing radiation nature is increasingly being used for ATFL diagnosis. However, diagnosis of ATFL injuries requires its segmentation from ultrasound images that is a challenging task due to the existence of homogeneous intensity regions, homogeneous textures and low contrast regions in ultrasound images. To address these issues, this research has developed an efficient ATFL segmentation framework that would contribute to accurate and efficient diagnosis of ATFL injuries for clinical evaluation.

**Methods:**

The developed framework comprises of five computational steps to segment the ATFL ligament region. Initially, region of interest is selected from the original image, which is followed by the adaptive histogram equalization to enhance the contrast level of the ultrasound image. The enhanced contrast image is further optimized by the particle swarm optimization algorithm. Thereafter, the optimized image is processed by the Chan–Vese method to extract the ATFL region through curve evolution; then the resultant image smoothed by morphological operation. The algorithm is tested on 25 subjects’ datasets and the corresponding performance metrics are evaluated to demonstrate its clinical applicability.

**Results:**

The performance of the developed framework is evaluated based on various measurement metrics. It was found that estimated computational performance of the developed framework is 12 times faster than existing Chan–Vese method. Furthermore, the developed framework yielded the average sensitivity of 98.3 %, specificity of 96.6 % and accuracy of 96.8 % as compared to the manual segmentation. In addition, the obtained distance using Hausdorff is 14.2 pixels and similarity index by Jaccard is 91 %, which are indicating the enhanced performance whilst segmented area of ATFL region obtained from five normal (average Pixels—16,345.09), five tear (average Pixels—14,940.96) and five thickened (average Pixels—12,179.20) subjects’ datasets show good performance of developed framework to be used in clinical practices.

**Conclusions:**

On the basis of obtained results, the developed framework is computationally more efficient and more accurate with lowest rate of coefficient of variation (less than 5 %) that indicates the highest clinical significance of this research in the assessment of ATFL injuries.

## Background

Ankle ligaments are the most common human joints affected by sports injuries, accidents, high ankle sprains and inflammation [1]. Generally, ligaments are robust and strong fibrous tissues that connect two bones in the ankle that are presented in the form of anterior talofibular, posterior talofibular and calcaneofibular and deltoid ligaments. Statistics shows that around 20 % of the professional sports persons are prone to injuries every year and around 14 % of the all sports injuries are related to ankle, out of which, 80 % injuries belong to ligaments [2, 3]. In addition, it was estimated that around 23,000 individuals in United States and 5000 individuals in Great Britain undergo daily treatment due to ankle injuries [4–6]. Because of the active life style and involvement in sports activities by individuals, the incidence of ankle injuries are growing rapidly and may reach up to 40 % [7–9].

**Fig. 1:**
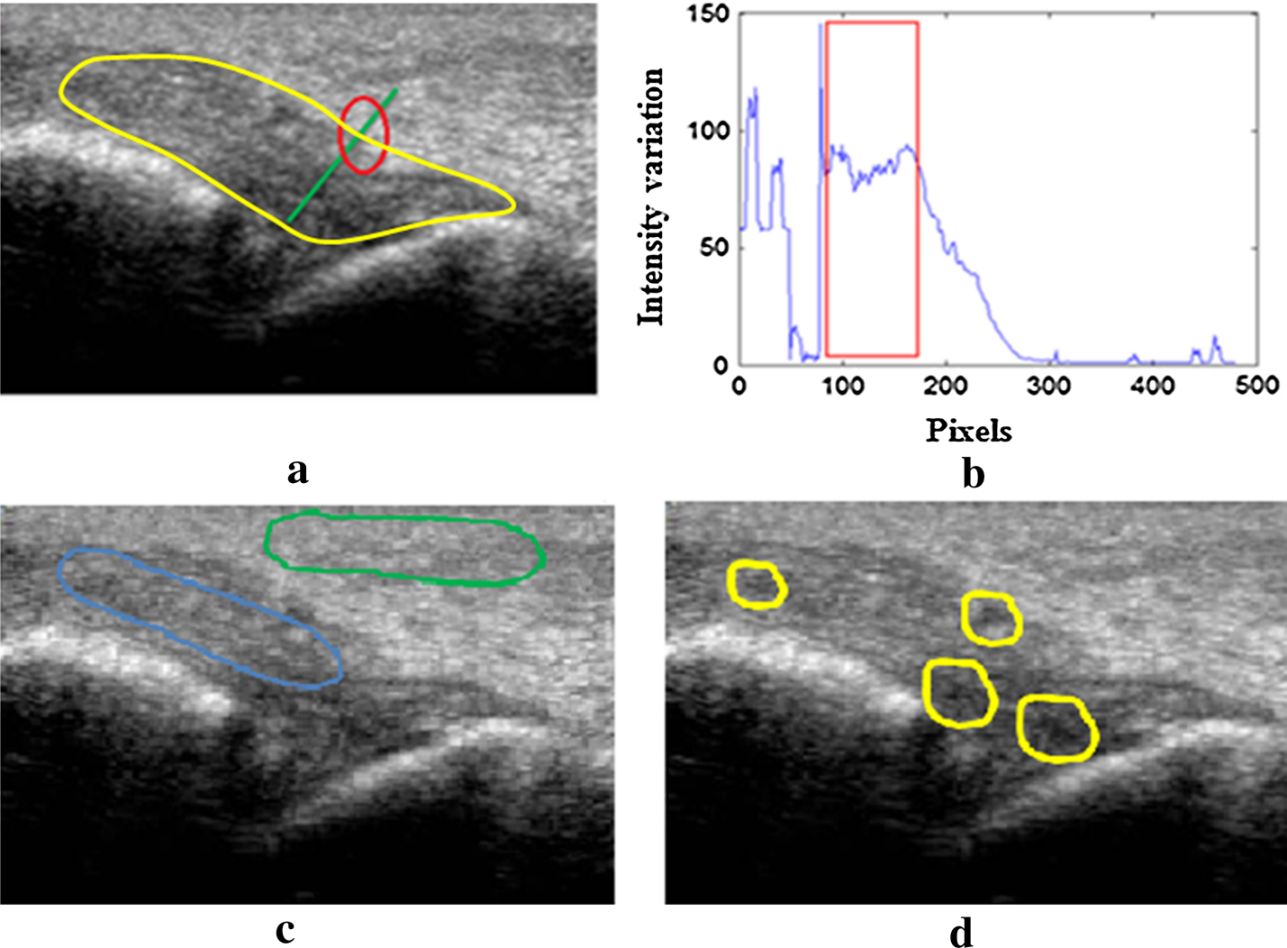
Challenges in ultrasound images of ATFL **a** ATFL anatomy and homogeneous intensity region, **b** corresponding intensity graph, **c** homogeneous textures, and **d** low contrast regions

Research [10–12] has reported that anterior talofibular ligament (ATFL) is considered as the weakest ligament exists in ankle, and therefore most commonly affected by the injuries. ATFL starts at the front part of lateral malleolus (LM) and reaches till taller neck in the ankle. Any injury in ATFL ligament requires an individual patient to visit clinic and perform an imaging examination to ensure the damages associated to it. Currently, Magnetic Resonance Imaging (MRI) has the ability to visualise damages to injured ATFL, but limited availability, high cost, patient discomfort, long examination time and in particular, patient with injury need to be inside MRI scanner are considered as the major limitations [13–15]. Alternatively, ultrasound imaging with its portable, non-invasive and non-ionizing radiation nature increasingly is being used for ATFL diagnosis [15–19]. However, due to the limited capability of 2D ultrasound images that exhibits several challenges such as low resolution in ATFL region, homogeneous intensities in surrounding regions of ATFL, homogeneous texture between ATFL and surrounding tissues and low contrast (see Fig. 1), only expert radiologist and orthopaedic surgeons are able to visually interpret the injured ATFL [20–22].

Due to the challenges associated with 2D ultrasound images of ATFL ligament, direct interpretation and visualization of injuries associated with injured ATFL is not recommended as it may lead to wrong diagnosis. This warrants the requirement of computational methods that are capable of delineating ATFL region from 2D ultrasound images [23, 24]. However, the development of computational methods to delineating ATFL region is further challenging due to the issues associated with 2D ultrasound images. These issues can be seen in 2D ultrasound images as homogeneous intensity region in ATFL and surrounding tissues that results in the difficulties to extract boundaries of ATFL region computationally as illustrated in Fig. 1a, b. In addition, Fig. 1a has indicated the location of ATFL ligament in yellow colour and homogeneous intensity region is presented in red circle and green line. Furthermore, computational methods might not be able to distinguish between ATFL and surrounding regions due to similar texture available in both regions as depicted in Fig. 1c in red and blue indicated regions. Finally, contrast within the ATFL region varies rapidly and may results in computational difficulties to identify the correct region of interest from 2D ultrasound images, which are presented by the yellow colour circles.

In order to deal with the challenges associated with 2D ultrasound images, several computational approaches were developed to segment different tissues using region based, edge based, thresholding based, wavelet based, pattern or texture classification based, atlas and deformable based methods and non-parametric probabilistic model with shape driven based methods [25–28]. Although, above stated methods are capable to segment tissues from 2D ultrasound images, fake edge detection, incompatibility with noise, under segmentation/over segmentation, variable shape, inconstant size and unpredictable properties are some of the known limitations with these methods [23, 29]. So far, the above methods were not utilised to segment ATFL region from 2D ultrasound images. In addition, due to the known limitations as listed above, it is not suggestive to use these methods to segment ATFL region as it exhibit complex nature in shape, size and texture. Thus, the main objective of this research is to develop a computational method for the segmentation and accurate visualisation of ATFL from 2D ultrasound images that will impact the clinical evaluation of ATFL injuries leading to betterment of quality of life in individuals.

In order to achieve the main objectives as stated above, a new segmentation framework for ATFL segmentation from 2D ultrasound images is developed in this research that includes five (5) step processes comprising of region of interest (ROI) initialization, adaptive histogram equalization (AHE) [30, 31], particle swarm optimization (PSO) [32], Chan–Vese method [33] and morphological operation [34–36] as sophisticated image processing and computational techniques. The ATFL segmentation framework is developed in order to resolve the issues associated with 2D ultrasound images of ATFL as depicted in Fig. 1a, b, c, d. In the developed framework, selection of ROI by visual interpretation followed by the automatic cropping of ROI is used to resolve the issue of homogeneous boundaries between ATFL and surrounding tissues. Applied AHE is a technique to enhance contrast is used to resolve the issue of low contrast in ATFL region as it has been found capable of producing better results than other contrast enhancement approaches in various image processing applications due to its suitability with variable contrast images [37]. Thereafter, the PSO algorithm is used in this framework to resolve the issue of high computational time and the suitability of this method have been demonstrated on natural and satellite image databases that shows significantly better outcomes [15–18]. The Chan–Vese method is applied in the developed framework to resolve the issues of low visibility and boundary extraction at ATFL region as it has been found suitable to segmented desired tissue area compared to traditional active contour and other existing segmentation methods, qualitatively and quantitatively [38–43].

The descriptive illustration with detailed approach is discussed in the next section followed by its capability to segment ATFL region from 2D ultrasound images. In the next sections, performance metrics are measured to demonstrate the performance of the developed framework followed by the results and discussion that shows the impact of the developed framework for clinical evaluation of ATFL injuries.

## Methods

### Image dataset

In this study, a video (length—3 s, 25 frames/second) of the ATFL region is acquired using linear probe (5–13 MHz) of iU22 Philips colour ultrasound machine from 25 subjects (12 healthy, 8 patients with tear injury and 5 with thickened ligament injury) with the age ranges from 18–60 (mean 34) years. Institutional medical ethics approval was obtained prior to the study. Subjects were informed about the study protocol and consent form is obtained from all the subjects. A total of 1250 slices are extracted in the video acquired from 25 subjects, each video contains 75 slices representing ultrasound images of ATFL region. The involved three experts have the experience of 18 years, 9 years and 5 years in ultrasound imaging are asked to visually inspect 75 slices of each dataset and assign a grade (lowest 1, low 2, moderate 3, high 4, and highest 5) to each slices based on the quality according to the method proposed earlier [44]. In most datasets, around 36–37 slices are graded as score 5, around 13–14 slices are graded as score 4, around 9–10 slices are graded as score 3, around 5–6 slices are graded as score 2 and remaining are graded as score 1. In this study, slices those were graded as 4 and 5 are included for the further processing and post processing is performed on 50 slices of each subject.

### ATFL segmentation framework

**Fig. 2:**
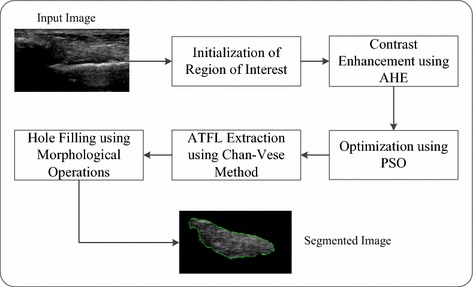
The developed segmentation framework for ankle ATFL ligament

As discussed in introduction section of this paper, this research is mainly focuses on three major challenges such as homogeneous intensity, homogeneous texture and low contrast regions that create the difficulties in ATFL interpretation and extraction from ultrasound images. To overcome these problems, a framework is developed to segment the ATFL region from ultrasound images as illustrated in Fig. 2.

As presented in Fig. 2, there are mainly five steps involved in the segmentation of ATFL from 2D ultrasound images. At first, process of segmentation starts with the initialisation of ROI in ultrasound image frame consisting of ATFL region, which is followed by the AHE method to increase the contrast of the ROI. The contrast enhanced image is further processed by the PSO algorithm for optimization to produce more accurate results. In the following steps, the Chan–Vese method is applied on the optimized image to extract the desired ATFL region. The extracted images are smoothed by the morphological operation for better visualization and accurate interpretation. It should be noted that this research has developed a novel segmentation framework due to the unique hybridization of advanced image processing and optimization algorithms for ATFL segmentation for the first time. The details of the developed framework are described in the following sections:

#### Initialization of ROI

**Fig. 3:**
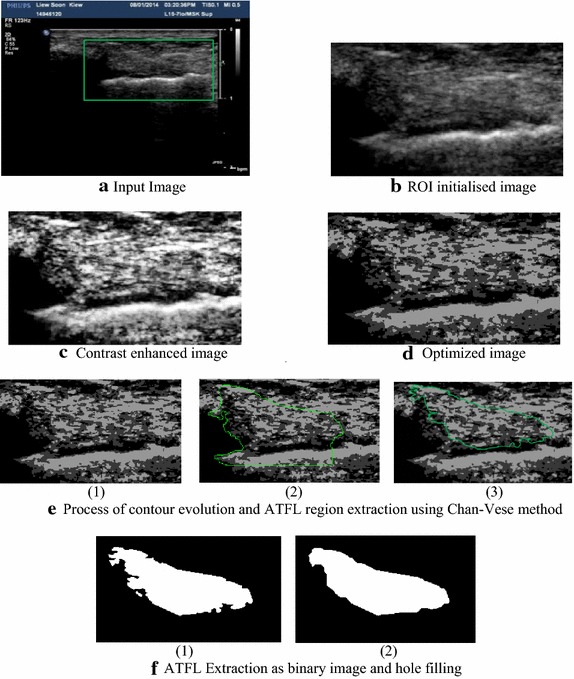
Steps showing ATFL segmentation using developed framework: **a** input 2D ultrasound image of ATFL region, **b** ROI initialised image after visual inspection by experts, **c** contrast enhanced image after applying adaptive histogram equalisation, **d** optimized image as a result of PSO, **e** process of contour evolution and ATFL extraction using Chan–Vese method (1–2–3) and, **f** ATFL extraction as binary image and hole filling (1–2)

In this research, ROI is initialized prior to post-processing due to the followings reasons: (1) simplifying the input image, (2) reducing the occurrences of errors during ATFL extraction and, (3) better computational performance. For the selection of ROI, variability of ATFL location in ultrasound images is considered and expert radiologist inspected ATFL region in several slices. In this framework, ROI is selected by selecting a defined region of interest that includes presence of ATFL within this region and an automated cropping of selected region is made. An example of ROI selection where ROI initialized region is indicated by green colour rectangle on input image is shown in Fig. 3b which is extracted from original image as shown in Fig. 3a. Once the ROI selection is performed, developed framework utilises selected ROI to perform further operations as discussed in the next following sections.

#### Contrast enhancement using AHE

Contrast enhancement methods such as histogram equalization are widely used in image processing to improve the interpretation capability of the resultant image. However, traditional histogram equalization does not performing well for variable contrast images [30]. In such cases, adaptive histogram equalisation (AHE) can be used that shows better performance to adjust the local contrast and deals better with variable contrast [31]. Thus, AHE is used in this research that may provide better outcomes than traditional histogram.

Mathematically, AHE can be described in Eqs. 1 and 2 [30, 31]:

1 $$\hat h\left( {m,n,g} \right) = \delta {\left( {g,x\left( {m,n} \right)} \right)^{m,n}}\cdot{f_w}\left( {m,n} \right)$$

2 $${f_w}(m,n) = \left\{ {\begin{array}{*{20}{c}} {{w^{ - 2}},\begin{array}{*{20}{c}} {\left| m \right| \le (w - 1)/2,}&{\left| n \right| \le (w - 1)/2} \end{array}}\\ {0,\begin{array}{*{20}{c}} {}&{}&{}&{\begin{array}{*{20}{c}} {\begin{array}{*{20}{c}} {}&{} \end{array}}&{}&\,\,\,\,\,\,\,\,\,\,\,\,\,\,\,\,{otherwise}&{} \end{array}} \end{array}} \end{array}} \right.$$

where, *x* is an input image, $\hat{h}$ presents the histogram and *δ* is the Kronecker delta function. *g* indicates an image with grey levels. The parameter *f*_*w*_ indicates the rectangular kernel with *m* rows and *n* columns, and *w* represents the width of the window. In this research, size of the kernel is considered as equal to the size of input image to cover all the objects. The ROI initialized image shown in Fig. 3b is further improved by the AHE method that helps in the betterment of contrast of input image as shown in Fig. 3c.

#### Optimization using PSO

PSO algorithm optimizes a problem iteratively to find out the candidate solution by moving particles around in search space. Each particle has a specific position and velocity. The corresponding position *x*_*i*_(*t*) and velocity *v*_*i*_(*t*) are determined and update based on the Eqs. 3 and 4 [32, 45–47]:

3 $$v_{i} \left( {t + 1} \right) = wv_{t} \left( t \right) + c_{1} r_{1} \left( t \right)\left( {y_{i} \left( t \right) - x_{i} } \right) + c_{2} r_{2} \left( t \right)\left( {\hat{y}\left( t \right) - x_{i} \left( t \right)} \right)$$

4 $$x_{i}^{{}} (t + 1) = x_{i}^{{}} (t) + v_{i}^{{}} (t + 1)$$

where, *w* represents the inertia weight that provides the storage to resultant velocities, $y_{i} \left( t \right)-x_{i}$ works based on each particle personal experience with respect to the best solution and $\hat{y}_{i} (t) - x_{i}$ indicating the confidence of whole swarm for best solution. The *c*_1_ and *c*_2_ are acceleration constants that are used to speed-up the image particles, $\left\{ {r_{1} \left( t \right),r_{2} \left( t \right)} \right\} \sim u\left( {0,1} \right)$, in which *u*(0,1) represents a random number range from 0 to 1. The *t* indicates the time unit. The *x*_*i*_(*t*) and *v*_*i*_(*t*) represents the current position and velocity of a particle *i* at time *t*. The term *y*_*i*_(*t*) indicates the personal best position and $\hat{y}_{i}$ is the global best position of a particle *i*.

PSO is a problem-independent algorithm, which means that little specific knowledge relevant to given problem is required. What we have known is just the fitness evaluation for each solution. This advantage makes PSO more robust than many other search algorithms. The main benefits of the PSO algorithm as compared to existing genetic algorithm and other heuristic algorithms in image segmentation are as follows: (1) The PSO algorithm is easy to implement and only few parameters have to be adjusted, (2) unlike the genetic algorithm, the PSO algorithm has no evolution operators such as crossover and mutation, which can be the main cause of computational complexity in some cases, (3) in PSO, only global best particle gives out information to the other image particles rather than whole population that makes it more robust, (4) unlike other heuristic algorithms, PSO has the flexibility to control the balance between global and local exploration of the search space.

Basically, the PSO algorithm is a multilevel thresholding approach that optimizes the energy level of the input image and helps in efficient curve evolution to extract the desired ATFL ligament region. In image segmentation, the use of PSO method is quite simple and effective, which improves the real-time performance of the image segmentation to a large extent. This research performs the segmentation on ATFL ligament ultrasound images based on the PSO algorithm to optimise the energy level of the image as illustrated in Fig. 3d. The experiments of segmentation indicates that the developed framework can get ideal segmentation results with less computation cost due to the efficient use of PSO algorithm. Therefore, the optimized image would help in efficient extraction of the ATFL boundaries by the curve evolution, which is performed by the Chan–Vese method as discussed in the next section of this paper.

#### ATFL extraction using Chan–Vese method

In this research, curve evolution is performed by the Chan–Vese method. The Chan–Vese method is the association of the Mumford–Shah mathematical model, level set method and curve evolution for energy minimization, accurate initialization and curve evolution during segmentation progression, respectively. The entire processing of the Chan–Vese method is described in Eq. 5 [33, 48–50]:

$$E^{CV} (c_{1} + c_{2} ,\varphi ) = E^{FT} (c_{1} + c_{2} ,\varphi ) + \mu L(\varphi )$$

5 $$= \left( {\int_{\varOmega } {|\text{Im} - c_{1} |^{2} H(\varphi )dxdy + } \int_{\varOmega } {|\text{Im} - c_{2} |^{2} (1 - H(\varphi ))dxdy} } \right) + \mu \int_{\varOmega } {\delta (\varphi )|\nabla \varphi |dxdy}$$

where, *c*_*1*_, *c*_*2*_ are constants and *φ* is an undefined curve. *E*^*FT*^represents the external energy function of curve *C*. *H*(*φ*) is heaviside function and *δ*(*φ*) is the Dirac one dimensional function. *L* is the image gradient and *μ* is a fixed parameter range less than 1. The image Im indicates the input image and the Im(*x*, *y*) depicts the particular image coordinate. Here ∇ stands for the gradient operator. In Eq. 5, function *E*^*CV*^(*c*_*1*_, *c*_2_, *φ*) depicted the Chan–Vese function.

Basically, the Chan–Vese method is the extended version of the traditional active contour method [51], which used in a variety of image processing tasks such as image segmentation and object boundary tracking. The traditional active contour method initially specifies a contour, which evolves under smoothness control (internal energy) and image driven forces (external energy) embedded with energy minimization capability to detect the boundary of the desired object. To minimize the energy, the traditional active contour method used Euler–Lagrange equation [52]. Although, this method is performing well, but it has some major limitations such as long runtime, need to initialize snake near to the object boundary and it is unable to merge two contours into one or split one contour into two contours. In order to overcome these issues, Chan and Vese proposed a segmentation method, which is applied in this research.

The Chan–Vese method does not depend on the edge function for the termination of shrinking or expanding curve for a preferred object boundary. The Chan–Vese method detects object boundaries much clearer; in case of undefined boundary gradients and noisy image. Thus, the Chan–Vese method is applied on the optimized images (e.g. PSO outcomes) to provide more accurate segmentation results as described in the developed framework. For example, boundary extraction of ATFL is performed by the Chan–Vese method [33] with 800 iterations as illustrated in Fig. 3e by three processing stages (1–2–3). Here, Optimized image is given as input for further processing using Chan–Vese method that shows number of iterations at different scales as shown in Fig. 3e and iterations are discontinued after its optimal position that leads to the extraction of ATFL region as shown by marked region in Fig. 3e, which is further extracted as binary image shown in left image in Fig. 3f.

#### Hole filling using morphological operation

Morphological operations are used to restore and reconstruct the damaged parts of the extracted image. Out of numerous morphological operations, this research applied the close operation to produce smoother results (see Fig. 3), which is described in Eq. 6 [34–36]:

6 $$C\left( {A,B} \right) = A \cdot B = E\left( {D\left( {A, - B} \right), - B} \right)$$

where, *C* is the close operator. *A* and *B* are the object sets of a binary image. *E* and *D* indicates the erosion and dilation, respectively. The close operation used two inputs such as, an image that need to be smoothed and a structuring element. However, this research used the disk structuring element to preserve the arbitrary shape of the object, which specifies a radius of 10 pixels so that the largest gap gets filled. For instance, during curve evolution the obtained segmented outcomes are not so accurate due to the asymmetrical boundaries and uncertain shape that leads to interpretation problem of the damaged tissues of ATFL as shown in left image of Fig. 3f. To overcome this problem, the developed framework uses morphological close operation on the extracted images for smoothing and clear boundaries as depicted in right image of Fig. 3f.

Figure 3 presents the entire process flow of ATFL segmentation performed by the developed framework. Figure 3a has shown the input ultrasound image of ATFL ligament, which presented the ROI initialization in green colour. Thereafter, the selected ROI is illustrated in Fig. 3b, which is further enhanced by the adaptive histogram equalization method to improve the contrast for better visualization of ATFL region (see Fig. 3c). The contrast enhanced image has shown the boundaries of ATFL ligament more clearly as compared to the input image. To make the enhanced image more optimized for further processing, the developed framework applied the PSO algorithm as depicted in Fig. 3d. The optimized image is further used in contour evolution, which is performed by the Chan–Vese method as illustrated in Fig. 3e by the three processing stages (1–2–3). In addition, Fig. 3e presented the process flow of the contour evolution to extract the ATFL region, which is indicated by the green colour. As mentioned earlier, the developed framework is capable to extract the ATFL region by the use of 800 iterations only, which are very less as compared to the existing methods. The extracted ATFL region is shown in Fig. 3f by two stages (1–2) that has the lacking of regular boundaries. To recover this issue, this framework used the morphological close operation to fill the gaps of boundaries for accurate interpretation of ATFL ligament injuries by the clinicians.

### Performance evaluation

**Fig. 4:**
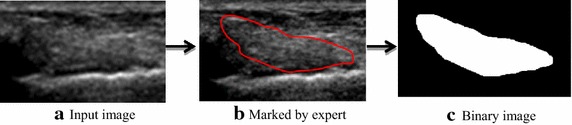
Manual segmentation of ATFL ligament by the expert

The developed framework was implemented on MATLAB [53, 54] running on a CPU (configuration: 64-bit operating system, 8.00 GB RAM, Intel (R) core (TM), i7-2600, 3.40 GHz). Once the developed framework is tested on the sample and image datasets, performance of the developed framework is further evaluated by measuring few performance metrics such as computation time, sensitivity, specificity, accuracy, Hausdorff distance, Jaccard index and segmented area which are further elaborated in the next sub-sections. After segmentation of ATFL region using developed method, performance metrics such as computation time and segmented area are directly measured from segmented images. However, for the measurement of sensitivity, specificity, accuracy, Hausdorff distance and Jaccard Index, the obtained segmentation results are compared with ground truths that were manually segmented from 2D ultrasound images by three expert radiologists having experience of 18 years, 9 years and 5 years. An example showing manual segmentation of ATFL region in 2D ultrasound image performed by the expert is illustrated in Fig. 4.

The reliability of above ground truths generation by different experts is estimated by the intra-observer variability among the experts. In order to measure the reliability of expert’s segmentation, measurement of true positive rate (TPR) among different experts is performed using Eq. 7 given in the next section.

### Performance metrics

In order to analyse the performance of the developed framework, computational time, sensitivity, specificity, accuracy, Hausdorff index, Jaccard index and segmented area metrics are explained in detail in the following sections [35]:

#### Computational time

Computational time is the amount of time for which a central processing unit (CPU) was used for processing instructions of a computer program that can be measured in seconds [55].

#### Sensitivity

Sensitivity is the proportion of true positives that are correctly identified by a diagnostic test. It shows how good the test is at detecting a disease [55].

7 $$Sensitivity = \frac{TP}{TP + FN}$$

where, true positive (TP) is the number of pixels correctly labelled as ATFL region, false negative (FN) is the number of pixels incorrectly labelled as non-ATFL region.

#### Specificity

Specificity is the proportion of the true negatives correctly identified by a diagnostic test. It suggests how good the test is at identifying normal condition [55].

8 $$Specificity = \frac{TN}{TN + FP}$$

where, true negative (TN) is the number of pixels correctly labelled as non-ATFL region, false positive (FP) is the number of pixels incorrectly labelled as ATFL region.

#### Accuracy

Accuracy is the proportion of true results, either true positive or true negative, in a population. It measures the degree of accuracy of a diagnostic test on a condition [55].

9 $$Accuracy = \frac{TP + TN}{TP + FP + FN + TN}$$

#### Hausdorff distance

Hausdorff distance is a validation metrics used in medical image segmentation for shape matching. Hausdorff index determined the degree of similarity between two superimposed sets, which is defined in Eq. 10 [56]:

10 $$H(A,B) = \max_{a \in A} \cdot \min_{b \in B} \left\| {a - b} \right\|$$

where, *H* presents the Hausdorff index. *a* and *b* are the points defined in sets *A* and *B*. ‖*a* − *b*‖ is indicating the underlying distance in tests.

#### Jaccard index

Jaccard index is a similarity measure that lies 0 to 1 as presented in Eq. 11 [56]:

11 $$J(A,B) = \frac{A \cap B}{A \cup B}$$

where, *J* depicts the Jaccard index, *A* indicates the region segmented by the developed framework and *B* is the region segmented by the experts.

#### Segmented area

The extracted desired and meaningful region from an input image is referred to as segmented area, which is determined by the calculation of number of pixels from the extracted region [55]. In this research, segmented area is calculated from normal and patients to demonstrates the clinical significant of the developed method.

## Results and discussion

### Segmentation of ATFL ligament

For the visual interpretation of segmentation processing of the developed framework and the corresponding outcomes with four sample images is illustrated in Table 1. For instance, initially, ROI is initialized on input image of sample 1 as shown in the 1st row and 1st column of Table 1 and obtained ROI image is presented by the 2nd row of this table. The selected ROI image is enhanced by the AHE method to increase the contrast level as shown in the 3rd row of Table 1 that helped in the better detection of ATFL boundaries as compared to the input image. This enhanced image is optimized by the PSO algorithm, which helps in accurate visualization of ATFL region inside the surrounding tissues as presented in 4th row, which is further processed by the Chan–Vese method to extract the ATFL region as illustrated in 5th row and the obtained segmentation result is shown in 6th row that need of smoothing to get the regular boundaries by morphological operations as depicted in 7th row of Table 1. The resultant of 7th row is further overlaid on the original image to show the region of ATFL with marked boundaries as shown in 8th row of Table 1. The obtained results are compared with manual segmentation performed by experts as shown in row 9th of this table whilst the binary images of experts segmentation is shown in row 10 of Table 1.

**Table 1:**
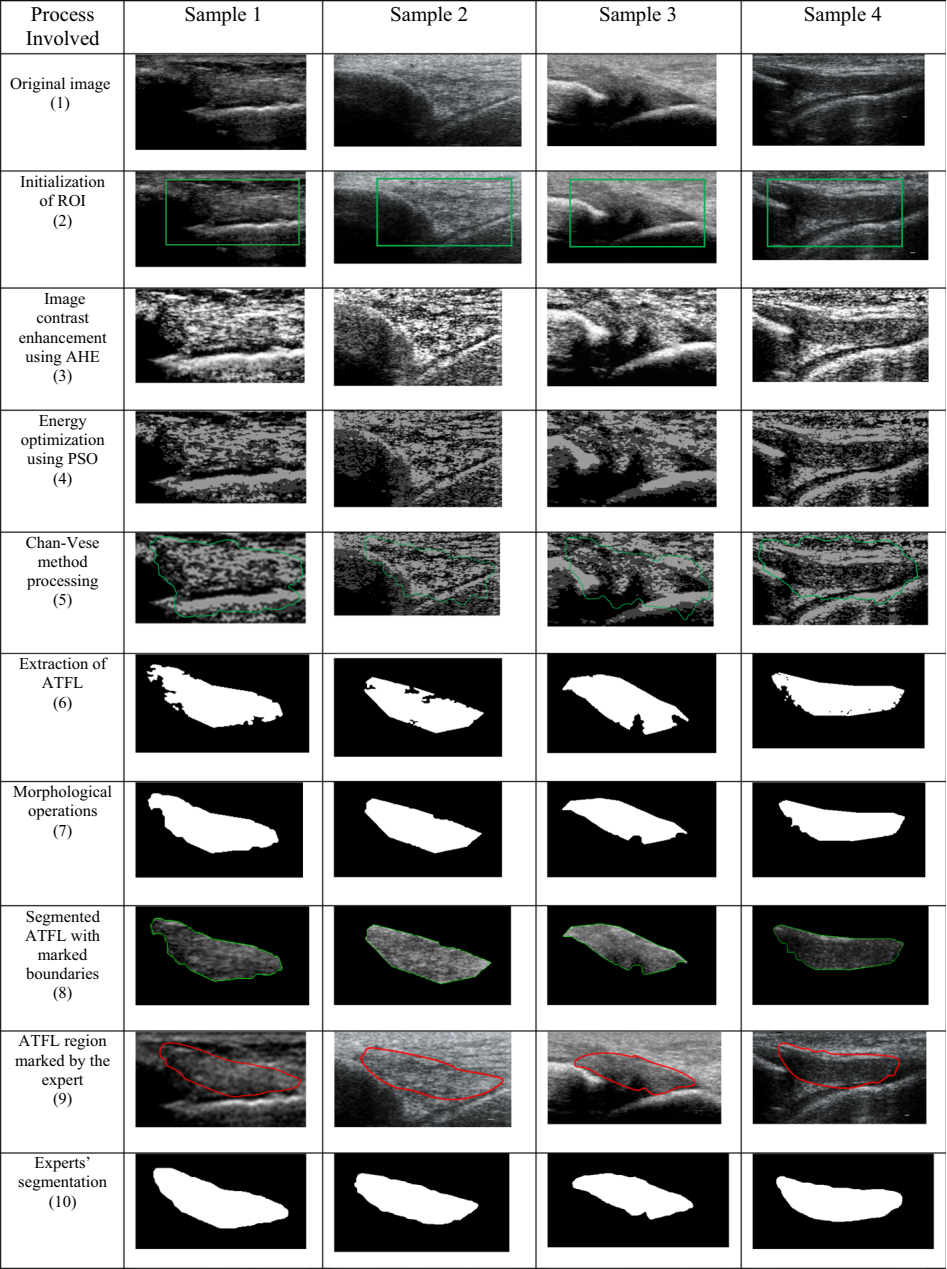
ATFL segmentation from 2D ultrasound images: steps involved (*first column*) and corresponding outcomes at each stages of developed framework for four different samples images (*column 2–5*)

### Performance evaluation

#### Computational time evaluation

In this section, performance of the developed framework is evaluated based on the estimation of computation time incurred using the Chan–Vese method and the developed framework at different number of iterations (e.g. 100, 500, 800, 1000, 2000 and 3000 iterations). The computational cost at different iterations is measured, which is illustrated in Table 2.

**Table 2:**
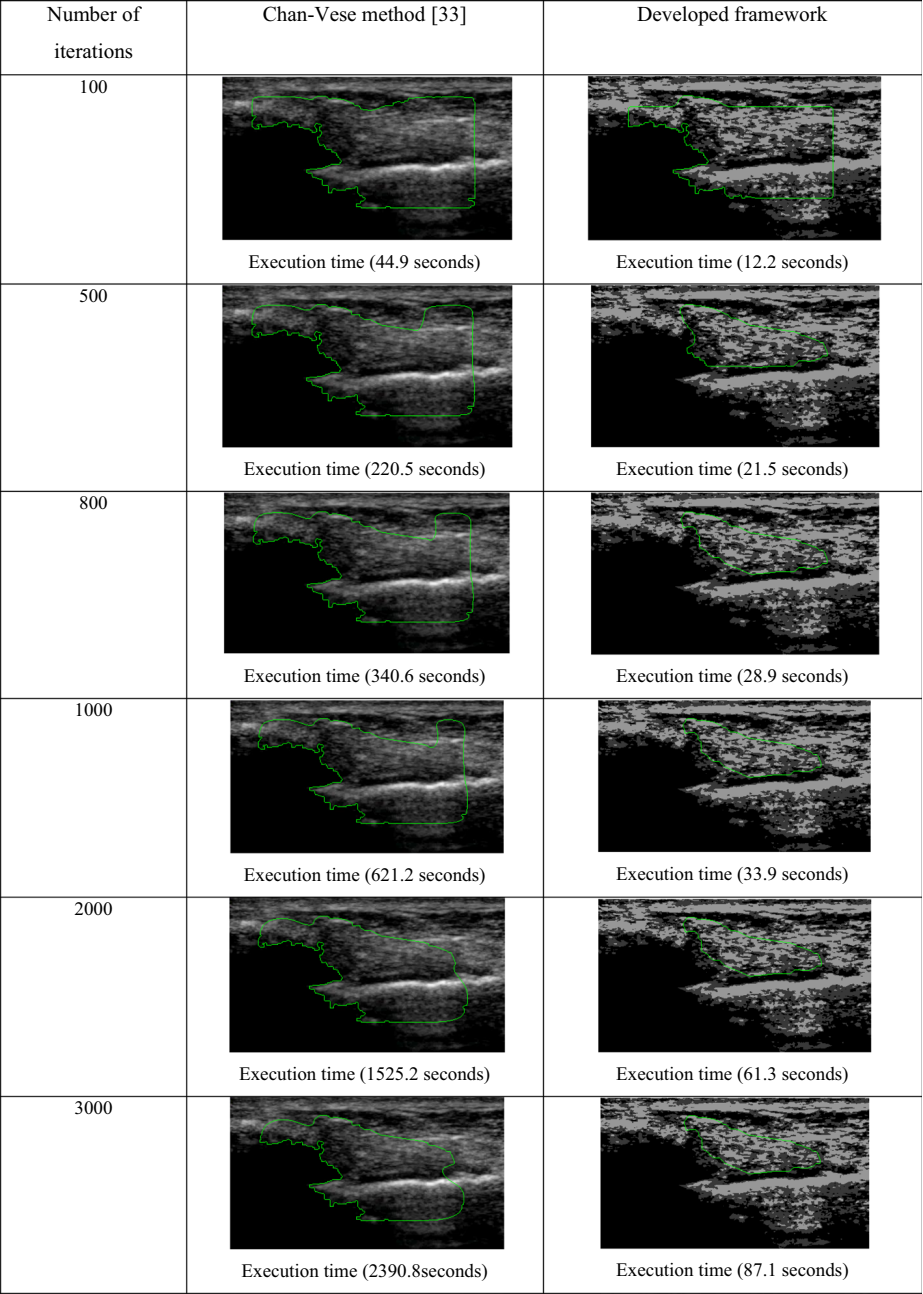
Computational performance evaluation of the developed framework

From Table 2, it can be clearly seen that even after 3000 iterations, the Chan–Vese method is not significant enough to track the ATFL region, while the developed framework leads to provide the optimal solution after only 800 iterations. After 800 iterations, no significant difference was found in tracked ATFL boundaries using the developed framework. Thus, 800 iterations are selected for all image segmentation in this research.

For the computational time, the developed framework yields marked improvement over the Chan–Vese method as the computational time taken by the developed framework (28.9 s) is approximately 12 times lower than the Chan–Vese method (340.6 s) for 800 iterations that results in optimal solution and performs faster than the existing Chan–Vese method.

#### Performance evaluation against the manual segmentation

The developed framework is tested for its performance whereby the obtained segmented ATFL results are compared with manually segmented ATFL from 2D ultrasound images by experts. Manual segmentation of ATFL from 2D ultrasound images were perform by three experts radiologist and the reliability of manual segmentation (considered as ground truth) is measured for all three experts. For the reliability, intra-observer variability among the experts is calculated as true positive rate of segmented area as discussed in material and method section of this paper. Therefore, the Table 3 illustrated the Intra-observer variability in the assessment of three experts for normal, tear and thickened ligaments.

**Table 3:** Intra-observer reliability calculated among three experts’ for normal, tear and thickened ligaments segmentation from 2D ultrasound images

	True positive rate estimation		
	Normal	Tear	Thickened
Expert 1–2	0.9345	0.9533	0.8765
Expert 2–3	0.8812	0.9268	0.9053
Expert 1–3	0.9165	0.9645	0.9434

As shown in Table 3, true positive rates calculated among expert 1 and expert 2 are significantly high for normal and tear ATFL regions that shows slightly less significant. At the same time, the estimated true positive rates among expert 1 and expert 3 are significant for all three datasets. However, true positive rates calculated among expert 2 and expert 3 are less significant compared to TPR calculated against Expert 1. The results show that Expert 1 has all comparable results expert 2 and expert 3. Thus, in this research, ground truths generated by Expert 1 are used to evaluate the performance of developed framework. The manual segmentation performed by Expert 1 are considered as ground truths and compared with results obtained using developed framework. The comparison is made as the measurement of sensitivity, specificity and accuracy and results obtained are listed in Table 4.

**Table 4:** Performance evaluation: sensitivity, specificity and accuracy measurements

Category of subjects	Patient ID	Type of used data	Sensitivity (%)	Specificity (%)	Accuracy (%)
Healthy subjects	1	Normal	98.7	96.3	96.6
	2	Normal	99.0	96.5	96.8
	3	Normal	99.0	96.4	96.8
	4	Normal	98.3	96.9	97.1
	5	Normal	98.9	96.6	97.0
	6	Normal	98.8	96.4	96.7
	7	Normal	99.3	96.6	96.9
	8	Normal	97.7	96.3	96.4
	9	Normal	99.2	96.3	96.7
	10	Normal	97.1	96.8	96.9
	11	Normal	97.7	96.8	96.9
	12	Normal	98.2	96.3	96.6
Subjects with injuries	13	Tear	97.6	96.8	96.9
	14	Tear	97.2	97.2	97.2
	15	Tear	95.7	97.1	96.9
	16	Tear	95.8	97.0	96.8
	17	Tear	98.0	96.6	96.8
	18	Tear	98.8	97.0	97.2
	19	Tear	99.6	96.3	96.8
	20	Tear	99.2	96.2	96.6
	21	Thickened	99.0	96.2	96.6
	22	Thickened	98.9	96.7	97.0
	23	Thickened	98.1	96.2	96.4
	24	Thickened	99.0	96.5	96.8
	25	Thickened	98.5	96.6	96.9
		Average	98.3	96.6	96.8
		Standard deviation	2.02	0.29	0.20
		Coefficient of variation	0.020	0.003	0.002

Table 4 presents the sensitivity, specificity and accuracy analysis for three different datasets used in this research. Analysis is performed on healthy and injured (such as tear and thickened patients) subjects ultrasound datasets. As shown in Table 4, the average sensitivity value obtained from the assessment of the developed framework results that are compared with manual segmentation performed by an expert is ranges from minimum 95.7 to maximum 99.6 % with an average sensitivity of 98.3 % (coefficient of variation—0.01 %). Similarly, specificity ranges from minimum 96.2 to maximum 97.2 % with an average specificity of 96.6 % (coefficient of variation—0.02 %). Furthermore, accuracy of the developed framework is evaluated that ranges from minimum 96.4 to maximum 97.2 % with the average accuracy of 96.8 % (coefficient of variation—0.01 %). The analysis based on the obtained results from sensitivity, specificity and accuracy have shown the significance and medical applicability of this research in clinical settings as the obtained accuracy is more than 96 %, standard deviation is less than 3 % and coefficient of variation is less than 5 %.

#### Performance evaluation based on the distance and similarity metrics

In this research, performance of the developed framework is not only be measured against ground truth segmentation, but also using performance metrics such as Hausdorff and Jaccard indexes that represents distance and similarity rate, which is compared with manually segmented ATFL results (see Table 5).

**Table 5:** Average similarity measure with Hausdorff and Jaccard indices

Distance and similarity measures		
Comparative analysis	Hausdorff index	Jaccard’s index
The developed framework versus experts	14.2	0.91
The Chen–Vese method versus experts [33]	19.2	0.71
Traditional active contour method versus experts [51]	41.3	0.42

The performance of the developed framework is compared against existing methods such as Chen–Vese and traditional active contour methods. As depicted in Table 5, compared to segmentation made by the experts, the distance/similarity metrics (Hausdorff and Jaccard) are indicated that the performance of the developed framework is more promising for ATFL segmentation than others as the similarity index measured by Jaccard metrics has got 91 % accuracy, which is higher than the traditional active contour (42 %) and the Chan–Vese method (71 %) accuracy rate. In addition, Hausdorff index value is achieved the lowest 14.2 than the others. The obtained metrics (Hausdorff and Jaccard) estimation has proven the strong validation of the developed framework.

Similarly, the average segmented area is measured from the results obtained from the developed framework, experts segmentation, traditional active contour method and the Chan–Vese method as shown in Table 6.

**Table 6:** Average segmented area and area ratio of the obtained results

Method	Segmented area (pixels)
The developed framework	16,941
Experts	17,045
The Chen–Vese method [33]	17,242
Traditional active contour method [51]	50,045

As illustrated in Table 6, segmented area determined based on the results produced by the experts is 17,045 pixels. However, calculated area from the traditional active contour method and Chan–Vese method are 50,045 and 17,242 pixels, respectively that are somehow far from the experts’ segmentation results. The area pixels calculated from the results obtained using developed framework is 16,941 pixels that shows the nearest values to the experts segmentation results and shows better performance than the existing methods.

#### Clinical significance

Since the developed framework works well for ATFL segmentation based on 2D ultrasound images and its performance evaluation shows high applicability in clinics. Next step is to measure the clinical significance of the developed framework. In this research, clinical significance of the developed framework is measured by evaluating three different kinds of subjects such as healthy subjects, injured ATFL comprises tear and thickened ligaments. In the first phase of evaluation to show clinical significance, the developed framework is applied on datasets containing normal, tear and thickened ATFL in ultrasound images and sensitivity, specificity and accuracy analysis is performed to demonstrate accuracy of developed framework not only for normal ATFL but also for injured ATFL. Table 7 presented the sensitivity, specificity and accuracy analysis for 12 normal, 8 tear and 5 thickened subject dataset.

**Table 7:** Clinical significance of the developed framework

Type of injury	Measures	Sensitivity (%)	Specificity (%)	Accuracy (%)
Normal (12 subjects)	Average	98.70	96.44	96.74
	Standard deviation	0.39	0.23	0.24
	Coefficient of variation	0.004	0.002	0.002
Tear (8 subjects)	Average	97.73	96.77	96.90
	Standard deviation	1.46	0.37	0.21
	Coefficient of variation	0.015	0.004	0.002
Thickened (5 subjects)	Average	98.49	96.52	96.78
	Standard deviation	0.69	0.22	0.19
	Coefficient of variation	0.007	0.002	0.002

As shown in Table 7, obtained average values of sensitivity, specificity and accuracy for normal, tear and thickened ATFL are more than 95 %. Therefore, the developed framework has performed well for Normal (sensitivity 98.70, specificity 96.44 and accuracy 96.74), tear (sensitivity 97.73, specificity 96.77 and accuracy 96.90) and thickened (sensitivity 98.49, specificity 96.52 and accuracy 96.90). As seen from the above table, it can be depicted that in all the measures, coefficient of variation is less than 5 % that shows the highest degree of significance of the developed framework for the clinical applications not only for normal ATFL evaluation but also for injured ATFL.

**Fig. 5:**
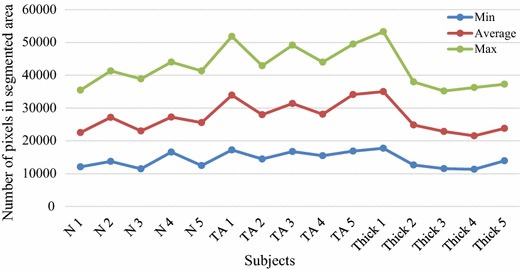
Assessment of segmented area for normal and injured ATFL ligaments

The clinical significance of the developed framework is also measured by estimating the total area of segmented region. In order to measure clinical significance by area analysis, five (5) datasets from each group of subjects are selected randomly. We have taken five normal subjects (indicated as N1, N2, N3, N4, and N5), five tear subjects (presented as TA 1, TA 2, TA 3, TA 4, and TA 5) and five thickened subjects (indicated as Thick 1, Thick 2, Thick 3, Thick 4 and Thick5) datasets. First, the developed framework is applied to segment ATFL region from all the datasets and from the resulting segmented ATFL region, pixel area measurement is performed for all datasets (as shown in Fig. 5).

As illustrated in Fig. 5, the graph presents the area pixel measured from the automatically segmented ATFL region using the developed framework. Here, the mean pixels obtained from ATFL region segmented from 2D ultrasound images of five normal subjects is 16,345.09 that shows higher values compared to the mean pixels obtained from ATFL region segmented in 2D ultrasound images of five tear (14,940.96) and five thickened (12,179.20) subjects’ datasets. As expected, normal ATFL have much bigger region compared to injured ATFL measurements. It should be noted that we have selected five subjects from each category due to the highest number of subjects in thickened category is five to maintain the consistency for this research. The developed framework is able to identify the normal and injured ATFL based on the average pixels and shows its applicability in clinical practices. Such measurements at the moment are not available. Thus, the developed framework can be used for the diagnosis of ATFL inquiries after a slight modification that can provide the user accessibility to directly important and perform all the steps involved in the developed framework.

## Conclusions

This research developed a novel framework to segment ATFL from 2D ultrasound images based on the integration of the ROI initialization, AHE, PSO, Chan–Vese method and morphological operation. The developed framework has marked a promising improvement over existing Chan–Vese method in terms of computational cost. In addition, distance/similarity, segmented area and area ratio metrics show the encouraging performance of the developed framework compared to existing methods. Since, ATFL segmentation from 2D ultrasound images has not been investigated earlier; this framework has opened new entrances for clinicians, radiologists, orthopaedists, rheumatologists and sports physician to visualize injuries and abnormalities of ATFL more accurately. In future, segmentation results can be used in 3D modelling of musculoskeletal tissues for better visualization and measurements. Hence, once the framework is bundled as a computer aided diagnosis (CAD) Tool, it would be able to assist the physician to diagnose ATFL disorders faster than the existing manual inspection methods by the clinicians.
